# Dry Eye Disease and personality: a systematic review

**DOI:** 10.3389/fmed.2026.1776417

**Published:** 2026-03-17

**Authors:** Alessandro Meduri, Emanuele Maria Merlo, Giorgio Sparacino, Laura De Luca, Maura Mancini, Giovanni William Oliverio, Paola Palino, Orlando Silvestro, Gabriella Martino, Pasquale Aragona

**Affiliations:** 1 Ophthalmology Clinic, Department of Biomedical and Dental Sciences and Morphofunctional Imaging, University of Messina, Messina, Italy; 2 Department of Biomedical and Dental Sciences and Morphofunctional Imaging, University of Messina, Messina, Italy; 3 Course Degree in Medicine and Surgery, University of Messina, Messina, Italy; 4 Department of Health Sciences, University Magna Graecia of Catanzaro, Catanzaro, Italy; 5 Department of Clinical and Experimental Medicine, University of Messina, Messina, Italy

**Keywords:** clinical psychology, DED, Dry Eye Disease, ophthalmology, personality, personality traits

## Abstract

**Background:**

Dry Eye Disease (DED) is a widespread condition associated with ocular discomfort and reduced quality of life. Personality traits may influence symptom perception, disease course and patients’ psychological adjustment, suggesting that subjectively experienced symptoms could be shaped by stable individual characteristics. This systematic review aimed to synthesize the available evidence regarding personality in individuals with DED.

**Methods:**

Following PRISMA guidelines, a systematic search was conducted in November 2025 in PubMed, Scopus and Web of Science using the terms “dry eye disease” OR “dry eye syndrome” AND “personality.” Inclusion criteria required full-text, peer-reviewed articles published in English and the use of standardized personality assessment tools. Studies were categorized according to whether DED was clinically confirmed or based on self-reported symptoms. Methodological quality was appraised using the NIH Study Quality Assessment Tools. The review protocol was registered in PROSPERO (CRD420251231024).

**Results:**

A total of 408 records were identified, and 8 studies met the inclusion criteria. Four studies involved clinically confirmed diagnoses of DED, while four relied on self-reported symptom measures. Across both groups, personality traits, particularly neuroticism and harm avoidance, were associated with greater symptom burden and reduced quality of life. Regression and mediation analyses in several studies supported the influence of personality on symptom perception.

**Conclusion:**

The findings suggest that personality traits contribute to the subjective experience and clinical impact of DED. However, the predominance of cross-sectional designs and reliance on self-reported measures limit causal interpretation. Further longitudinal and multimethod research is needed to clarify the underlying mechanisms and inform integrated clinical approaches that address both ocular and psychological aspects of DED.

**Systematic Review Registration:**

https://www.crd.york.ac.uk/PROSPERO/view/CRD420251231024, identifier PROSPERO (CRD420251231024).

## 1 Introduction

Dry Eye Disease (DED) is a multifactorial disorder characterized by a loss of homeostasis of the tear film and/or ocular surface, in which tear film instability and hyperosmolarity, ocular surface inflammation and damage, and neurosensory abnormalities are recognized as key etiological factors (1). DED is considered a multifactorial disorder in which dysfunction of the ocular structures responsible for producing and regulating tear film components leads to qualitative and/or quantitative deficiencies, resulting in tear film instability and hyperosmolarity (2). Several factors, including ocular and systemic diseases, topical and systemic medications, and environmental conditions, contribute to the onset and progression of DED (2–9). According to Papas and colleagues (10), the global prevalence of DED is estimated at 11.59%.

Given its worldwide diffusion and chronic course, DED significantly impacts patients’ quality of life (11–14), similarly to a wide range of ophthalmic conditions (15, 16). Quality of life is closely linked to psychological factors in chronic diseases, which frequently co-occur as comorbid conditions (17–25). Psychological dimensions are particularly relevant within ophthalmology (26). These include psychological influences on diagnostic outcomes (27–29), psychological factors contributing to the onset and maintenance of ophthalmic diseases (30–33), and psychological outcomes of ocular disorders (34–36).

Emerging evidence suggests that stress-related physiological pathways may also influence DED severity (37). Dysregulation of the hypothalamic–pituitary–adrenal (HPA) axis, alterations in autonomic balance, and increased release of pro-inflammatory cytokines have been associated with ocular surface inflammation and tear film instability. These mechanisms indicate a plausible biopsychosocial link, where chronic psychological stress may exacerbate symptom intensity and discomfort perception in DED.

In this context, personality traits become clinically relevant. According to influential studies, personality can be defined as a system of relatively stable individual differences in functioning, grounded in biological and psychological processes that shape patterns of affect, cognition and behavior (38–40). Personality characteristics should be differentiated from psychological states (38, 41–43). Traits are therefore understood as coherent dispositional characteristics capturing fundamental individual differences in personality (44, 45). State-related conditions, such as anxiety, depression and perceived stress, represent transient affective responses that may fluctuate over time, whereas personality traits reflect more stable individual predispositions. Both psychological states and personality traits lie on a continuum between typical functioning and psychopathology. While representing normative dimensions of individual variability, their abnormal and persistent expression may contribute to maladaptive patterns.

The subjective experience of DED may be modulated by psychological distress and neurosensory processing. Stable personality traits influence neural functioning by biasing the activity and regulation of brain networks implicated in stress responsivity, affective regulation, and interoceptive processing, thereby shaping patterns of neural connectivity and responsivity that contribute to the perception and modulation of chronic symptom experience (46, 47). Such modulation may involve trait-related differences in intrinsic connectivity within key circuits, including prefrontal, limbic, and attention networks influencing how individuals interpret, regulate, and persistently respond to ongoing physiological and psychological challenges. Among these traits, neuroticism, characterized by heightened emotional reactivity and negative affectivity, has been linked to amplified symptom reporting across a range of chronic conditions and is supported by neuroimaging evidence of trait-specific neural activity patterns (46, 47). A psychological perspective further suggests that personality traits modulate stress response systems and affective processing, contributing to sustained symptom burden through both cognitive-affective and physiological pathways (40). In this context, these mechanisms may explain the dissociation often observed between objective clinical signs and subjective symptom severity.

Personality influences how individuals perceive physical sensations, regulate emotions, respond to stress, and adhere to prescribed treatments. In chronic conditions characterized by persistent discomfort, such as DED, personality differences may shape symptom amplification, coping strategies, treatment adherence and quality of life. In accordance with the TFOS-DEWS-II definition, which emphasizes that ocular symptoms characterize DED, assessment of disease severity relies both on objective clinical evaluation and validated psychometric instruments. Validated psychometric instruments are designed to capture the subjective experience of symptoms. Instruments such as the Ocular Surface Disease Index (OSDI) (48), the 5-item Dry Eye Questionnaire (DEQ-5) (49), the Standard Patient Evaluation of Eye Dryness (SPEED) (50), the National Eye Institute Visual Function Questionnaire (NEI VFQ-25) (51) and the Impact of Dry Eye on Everyday Life (IDEEL) (52) are typically used to quantify symptom frequency, intensity and functional impact, as well as quality of life. As patient-reported outcomes measures (PROMs), these instruments provide standardized and reproducible data.

Several studies have already examined psychological and psychopathological variables associated with DED (53–61). For instance, Basilious et al. (53) and Han et al. (54) highlighted the prevalence of psychiatric and neurological conditions in individuals with DED, while He et al. (55), Liu et al. (56), Tang et al. (57), Tsai et al. (58), Vieira et al. (59), and Wan et al. (60) documented a robust association between affective symptoms and DED. These contributions have advanced the understanding of the psychological dimension with long-term implications for disease management and patient well-being.

However, despite these advancements, no systematic review has specifically investigated personality traits in individuals with DED. This represents a relevant gap, since personality is a relatively stable psychological dimension with potential long-term implications for disease management and patient well-being.

The present systematic review, therefore, aims to examine personality characteristics and dimensions as neuroticism, harm avoidance, anxiety-related traits such as anxiety sensitivity, extraversion, introversion, conscientiousness, low frustration tolerance and novelty seeking in individuals with DED. A clearer understanding of personality profiles associated with DED may support clinicians in identifying psychological factors contributing to symptom burden and in developing tailored, integrative, and more effective therapeutic approaches.

## 2 Methods

The present systematic review was conducted in accordance with the PRISMA guidelines for Systematic Reviews and Meta-Analyses and the PRISMA 2020 Checklist, to ensure methodological rigor and transparency (62–64). The review question was formulated using a PICO framework (Population, Exposure/Interest, Comparison, Outcomes) to define eligibility criteria and guide study selection (65). The review protocol was prospectively registered in the International Prospective Register of Systematic Reviews (PROSPERO): Registration Number: CRD420251231024.

### 2.1 Search strategy and information sources

PubMed, Scopus, and Web of Science were systematically searched between 1 August 2025 and December 2025 to identify relevant studies. The search strategy combined the terms “Dry eye disease” OR “Dry eye syndrome” AND “Personality” using Boolean operators to maximize sensitivity. “Personality” represents an all-encompassing term covering a wide range of related terms. The full search strings and keyword combinations used for each database are reported in Table 1. No restriction regarding publication year was applied. Only articles published in English, reporting original empirical data and specifically assessing personality in individuals with DED, were considered eligible. Editorials, reviews, conference abstracts, case reports, and non-peer-reviewed contributions were excluded.

**Table 1:** List of terms.

Number	Search terms	Combination
1	DRY EYE DISEASE	OR
2	DRY EYE SYNDROME	Combined as 1 or 2
3	PERSONALITY	AND
4	1 OR 2	
Final query	(1 OR 2) AND 3	–

Terms 1 and 2 refer to the ophthalmological diagnosis of interest, while term 3 refers to the psychological construct investigated. The final Boolean expression represents the combined search string used across databases.

### 2.2 Eligibility criteria

The inclusion criteria comprised full-text articles published in English in peer-reviewed, indexed journals, including participants with a diagnosis of Dry Eye Disease or clearly documented DED-related symptoms. Eligible studies were required to employ standardized psychodiagnostic instruments to assess personality traits, thereby reducing the risk of measurement bias. Given the observational design of the included studies, the presence of a traditional external comparison group was not required for eligibility. Systematic reviews and meta-analyses were also screened to identify additional relevant primary studies.

Exclusion criteria comprised conference abstracts, qualitative studies, narrative literature reviews, and case reports. Only empirical investigations using adequate statistical methodology and examining the relationship between DED and personality were considered eligible. Studies including mixed clinical samples, where the specific contribution of DED to personality outcomes could not be isolated, were excluded.

### 2.3 Primary outcomes

The primary outcome of this systematic review was the association between personality traits and DED symptoms. In accordance with the TFOS-DEWS definition, which recognizes ocular symptoms as central to DED, patient-reported symptomatology was considered as the clinical dimension to examine the influence of personality, focusing on associations between standardized personality traits and validated measures of dry eye symptom burden.

### 2.4 Selection process and data collection

**Figure 1:**
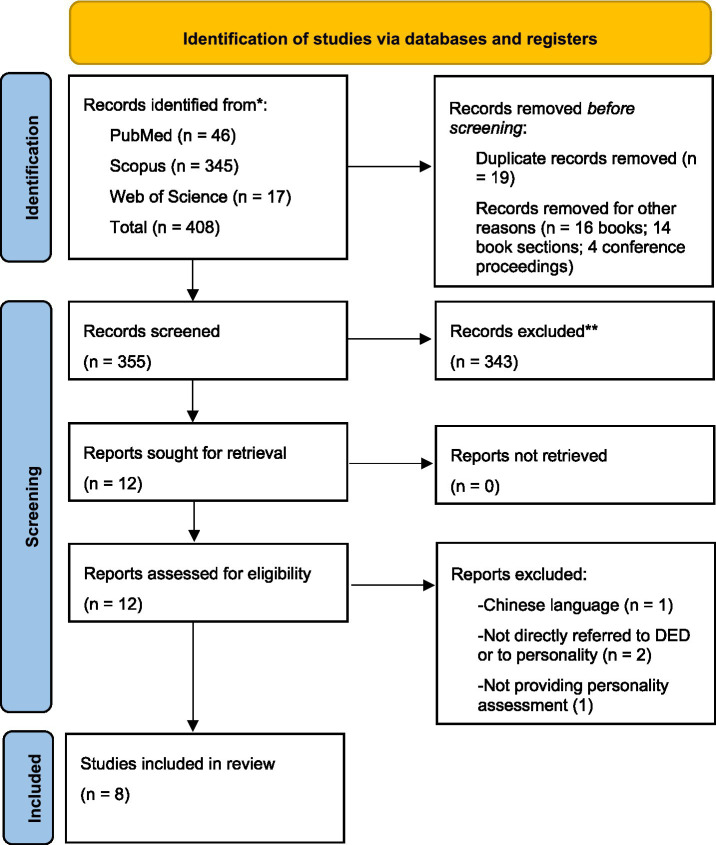
PRISMA flow diagram.

Two reviewers independently conducted the screening process. After removing duplicates, titles and abstracts were screened to identify studies that met the predefined inclusion criteria. Full-text articles of potentially eligible studies were then retrieved and assessed for eligibility. In addition, the lists of included studies and relevant systematic reviews were screened to identify further eligible contributions. A detailed overview of the study selection process is presented in Figure 1. For each included study, the following data were extracted: authors, publication year, country, study design, sample characteristics, DED assessment or clinically confirmed diagnosis, personality assessment instruments, and main findings. The extracted data are summarized in Table 2. Evidence was subsequently organized according to thematic domains identified across the included studies.

**Table 2:** Main characteristics of the included studies.

Authors	Year	Country	Study design	Sample	DED assessment/clinically confirmed diagnosis	Personality assessment	Main findings
Feroze et al. (66)	2020	Saudi Arabia	Cross-sectional	613 subjects 57% of the patients reported dry eye symptoms	Ocular surface disease index (OSDI)/not given, electronically distributed	Big Five Personality Inventory (BFI)	57.7% of the involved patients reported DED symptoms. Another significant correlation emerged between neuroticism and DED (*p* < 0.01)
Ichinohe et al. (67)	2016	Japan	Cross-sectional	56 DED patients	Ocular Surface Disease Index (OSDI)/Diagnosed	Big Five Personality Inventory (BFI)	Correlational analyses among objective signs, subjective symptoms and personality traits were performed. Significant correlations emerged between DED symptoms, Ocular Surface Disease Index and neuroticism (*p =* 0.008; *p =* 0.039). An influence of personality on subjective symptoms was suggested
Kaiser et al. (68)	2019	Germany	Cross-sectional	64 DED patients	Ocular Surface Disease Index (OSDI)/Diagnosed	Munich Personality Test (MPT)	Depression, well-being, resilience and personality traits were assessed among patients. In DED patients, depressive symptoms (affecting significantly 61% of patients) negatively correlated with the premorbid personality trait extraversion (*p =* 0.036) and frustration tolerance (*p =* <0.001). Positive correlations emerged between depression, premorbid neuroticism (*p =* 0.001), isolation tendencies (*p =* 0.014) and esoteric tendencies (*p =* 0.001)
Kuru et al. (69)	2023	Turkey	Cross-sectional	67 DED patients	Ocular Surface Disease Index (OSDI)/Diagnosed	Temperament and Character Inventory (TCI)	Temperament and character were studied in DED patients. Novelty seeking was lower than general population (*p* < 0.001), while harm avoidance and persistence were higher (*p* = 0.014; *p* < 0.001). A significant and positive correlation was found between harm avoidance and OSDI, while novelty seeking was in a negative relationship. Anxiety mediated the relation between novelty seeking, harm avoidance and OSDI score
Magno et al. (70)	2025	Norway Netherlands New Zealand United Kingdom	Cross-sectional	8.7% of the entire cohort (78,610 from the Lifelines cohort)	Women’s Health Study (WHS) Dry Eye Disease Questionnaire/ DED definition achieved by the used instrument	NEO Personality Inventory questionnaire (NEO-PI)	According to the fully corrected model including all demographic variables and comorbidities, higher rates of neuroticism were associated with higher odds of DED (OR 1.10, *p* < 0.001). On the contrary, conscientiousness was associated with lower odds of DED (OR 0.97, *p* = 0.014). Referring to high symptomatic DED, associations were strong (OR 1.23, *p* < 0.001, neuroticism; 0.90, *p* < 0.001), conscientiousness. Finally, greater extraversion was associated with lower DED symptom presentation (OR 0.93, *p* = 0.031)
Tananuvat et al. (71)	2022	Thailand	Cross-sectional	100 DED patients	Ocular Surface Disease Index(OSDI)//Diagnosed	15-item Neuroticism Inventory (NI-15)	Neuroticism was studied together with perceived stress and quality of life in DED patients. Neuroticism strongly predicted decreased quality of life and increased distress among patients (*p* < 0.05)
Toth et al. (72)	2020	Croatia	Cross-sectional	381 community-based subjects 22.8% of the participants reported dry ocular surface according to the Ocular Surface Disease Index-OSDI threshold	Ocular surface Disease Index (OSDI)/Not given, online survey methodology	Anxiety Sensitivity Index (ASI)	Anxiety sensitivity was studied as a personality trait. Women reported more dry eye symptoms considerable as moderate or severe. Anxiety sensitivity, with major reference to psychological concerns, predicted dry eye symptom intensity (*p* < 0.01)
Toth et al. (73)	2025	Croatia	Cross-sectional	776 subjects 63.97% of the participants ranged from mild to severe dry eye according to the Ocular Surface Disease Index-OSDI threshold	Ocular Surface Disease Index (OSDI)/ Not given, online survey methodology	Anxiety Sensitivity Index (ASI)	Anxiety sensitivity was assessed alongside measures of state depression, anxiety, and stress Significant and positive correlations emerged between anxiety sensitivity, emotional states and ocular surface status (*p* < 0.01). Highly ranking anxiety sensitivity individuals were more disposed to experience and perceive DED symptoms

ASI, Anxiety Sensitivity Index; BFI, Big Five Inventory; DED, Dry Eye Disease; MPT, Munich Personality Test; NEO-PI, NEO Personality Inventory; NI-15, 15-item Neuroticism Inventory; TCI, Temperament and Character Inventory.

### 2.5 Risk of bias assessment

The methodological quality of the included studies was independently assessed by two reviewers, using the NIH Study Quality Assessment Tools, selecting the appropriate checklist according to each study’s design. Any discrepancies were discussed until a consensus was reached. The overall risk of bias evaluation contributed to the interpretation and the qualitative synthesis of the findings.

## 3 Results

The search process is illustrated in Figure 1. A total of 408 records were initially identified. After removing 19 duplicates, 389 records remained. Of these, 355 records were excluded based on title and abstract screening stage, while 34 were excluded because they did not meet the eligibility criteria. The reference lists of potentially relevant articles and related systematic reviews were also screened. A total of 12 full-text articles were retrieved and assessed for eligibility. Among these, one article was excluded due to publication language (Chinese), two did not specifically concern Dry Eye Disease, and one did not employ a standardized personality assessment tool. Ultimately, eight studies met all inclusion criteria and were included in the review. The main characteristics and extracted data of the included studies are reported in Table 2.

### 3.1 Characteristics of the included studies

Across the eight included studies, all employed cross-sectional designs (66–73). The studies were relatively recent, published between 2016 and 2025. Geographically, one study was conducted in Saudi Arabia (66), one in Japan (67), one in Germany (68), one in Turkey (69), one involved participants from Norway, the Netherlands, New Zealand and the United Kingdom (70), one was conducted in Thailand (71), and two in Croatia (72, 73). Regarding DED assessment, four studies combined validated dry eye questionnaires with ophthalmic examination, allowing a clinically confirmed diagnosis of DED (67–69, 71). The Ocular Surface Disease Index (OSDI) was used in 4 studies in conjunction with clinical evaluation. The remaining four studies relied on remotely administered questionnaires, either through electronic distribution (66) or online survey methodology (70, 72, 73). Three of these used the OSDI without in-person clinical evaluation, while Magno et al. (70) employed the Women’s Health Study (WHS) Dry Eye Disease Questionnaire, which includes an item referencing a previously completed DED diagnosis. In these four studies, clinical confirmation of DED by direct ophthalmic examination was not available. Personality assessment instruments varied across studies. Two studies used the Big Five Personality Inventory (BFI) (66, 67), one used the Munich Personality Test (MPT) (68), one employed the Temperament and Character Inventory (TCI) (69), one used the NEO Personality Inventory questionnaire (NEO-PI) (70), one the 15-item Neuroticism inventory (NI-15) (71) and two used the Anxiety Sensitivity Index (ASI) (72, 73). Overall, personality was assessed using a variety of validated psychometric tools; however, heterogeneity in measurement instruments and variability in the certainty of DED diagnosis (clinical vs. self-reported) should be acknowledged when interpreting the findings.

### 3.2 Clinically confirmed diagnosis of Dry Eye Disease

Studies included in this section define Dry Eye Disease using clinically confirmed diagnostic criteria, combining objective ocular surface assessments with validated symptom questionnaires. Consistent with the TFOS-DEWS definition, the presence of ocular symptoms was a necessary diagnostic component, while clinical tests were used to support the diagnosis and characterize disease severity.

Four of the included studies provided a clinically confirmed diagnosis of DED in addition to validated symptom assessment instruments. Ichinohe et al. (67), examining 56 clinically diagnosed DED patients, administered both the OSDI and the DEQS. Their findings showed significant positive associations between neuroticism and DED symptom severity. Multiple regression analyses further indicated that neuroticism significantly predicted higher symptom burden, negatively affecting both quality of life and ocular discomfort indices.

Kaiser et al. (68) assessed personality in 64 patients with DED using the Munich Personality Test (MPT). Correlational analyses revealed negative associations between premorbid extraversion and frustration tolerance, suggesting that lower extraversion was associated with greater frustration tolerance. Conversely, premorbid neuroticism, isolation, and esoteric tendencies were positively correlated with depressive symptoms.

Kuru et al. (69), using the Temperament and Character Inventory (TCI) in 67 clinically confirmed DED patients, reported lower novelty seeking and higher harm avoidance and persistence relative to normative population benchmarks. Harm avoidance was positively correlated with DED symptom severity (OSDI scores), whereas novelty seeking showed a negative association. Mediation analyses suggested that anxiety partially mediated the relationship between temperament dimensions and symptom intensity. Similarly, Tananuvat et al. (71), administering the NI-15 and OSDI to 100 clinically diagnosed DED patients, found that neuroticism significantly predicted greater distress and reduced quality of life.

Across these studies, significant associations emerged between personality traits and DED symptom burden. Despite the use of different personality assessment instruments, findings consistently highlighted the relevance of neuroticism and harm avoidance as correlates of symptom severity. The inclusion of regression and mediation analyses in two studies (67, 71) further strengthens the evidence supporting a role of personality factors in shaping symptom perception and psychological functioning in DED.

### 3.3 Self-reported Dry Eye Disease symptoms

Studies included in this section identified Dry Eye Disease primarily based on patient-reported symptoms, using validated questionnaires and predefined cutoffs to classify symptom presence and severity. This classification reflects differences in case ascertainment methodology rather than a conceptual difference from clinically confirmed DED, which is by definition a symptomatic condition.

Four studies did not include an ophthalmologic examination to confirm the diagnosis of DED. In these cases, DED was identified through self-reported symptom measures, primarily the Ocular Surface Disease Index (OSDI) or the Women’s Health Study Dry Eye Disease Questionnaire (WHS), which also allows symptom grading (mild, moderate, or severe).

Feroze et al. (66) reported a significant correlation between neuroticism and DED symptom severity. Neuroticism and symptoms were assessed using the BFI and the OSDI in an electronically distributed survey.

Notably, 57% of participants reported DED-related symptoms, and the authors highlighted that this was the first study exploring the role of personality in DED within their national context.

Magno et al. (70), analysing a large population-based sample of 78,610 participants, identified a DED prevalence of 8.7% through the WHS questionnaire. Higher neuroticism scores were associated with greater symptom severity, while higher conscientiousness was linked to fewer symptoms. Greater extraversion was also associated with reduced symptom burden.

Toth et al. (72, 73) examined the relationship between anxiety sensitivity (treated as a personality trait) and self-reported DED symptoms using online survey administration. The first study found anxiety sensitivity to be more prevalent in women and significantly related to psychological concerns, predicting symptom severity. The second study demonstrated that anxiety sensitivity was specifically associated with perceived ocular surface discomfort. However, both studies were based on self-report measures and did not include clinical diagnostic confirmation.

Although symptom-based screening instruments such as the OSDI and WHS are validated and widely used in research and clinical practice, the TFOS-DEWS framework emphasizes that Dry Eye Disease is primarily defined by ocular symptoms, while clinical tests provide complementary information for disease characterization.

## 4 Discussion

Personality represents a relevant psychological domain in the study of individuals with medical conditions (74–80). While several psychological factors have been investigated in ocular surface disorders (81–84), the role of personality in Dry Eye Disease (DED) has received comparatively limited attention. The small number of studies specifically addressing this association highlights a gap in the current literature.

The findings of the present systematic review suggest that personality traits are involved in patients’ overall functioning and symptom experience in DED. Two main patterns emerged when distinguishing between studies with clinically confirmed DED diagnosis and those relying solely on self-reported symptoms. Although the results converge in several aspects, some interpretative considerations are warranted.

McMonnies (85) previously noted a potential discrepancy between subjective symptoms and objective clinical signs in DED. According to the author, elevated symptom severity in the absence of corresponding ocular surface abnormalities may be partly influenced by psychological and personality variables, including anxiety, distress, hypochondriasis, mood disturbances and, most consistently, neuroticism. Neuroticism has been broadly associated with heightened symptom perception, increased emotional reactivity, and reduced resilience. McMonnies referenced only one study (67), underscoring the scarcity of research on this topic; however, the present review expands this evidence base and confirms the centrality of personality factors.

A plausible interpretative framework linking personality and DED symptom perception involves the interaction between ocular surface physiology and stress-processing systems. Personality traits may influence the experience of DED symptoms through both biological and psychological pathways (86). Consistent with the results of the included studies, traits such as neuroticism, harm avoidance, frustration tolerance, novelty seeking and anxiety sensitivity showed significant associations with DED symptoms.

Five studies included in the results considered neuroticism (66–68, 70, 71). At the biological level, higher neuroticism has been associated with altered stress responsivity, including dysregulation of the hypothalamic–pituitary–adrenal axis, increased sympathetic activation, and enhanced inflammatory signaling (40, 46). These mechanisms have been implicated in ocular surface inflammation, tear film instability, and nociceptive sensitization, all of which are clinically relevant to the pathophysiology of Dry Eye Disease (1). At both cognitive and behavioral levels of functioning, individuals high in neuroticism tend to exhibit increased attentional focus on bodily sensations, negative symptom appraisal, and reduced tolerance for discomfort, which may amplify symptom awareness and reporting (39). Taken together, these converging pathways suggest that neuroticism may act as a vulnerability factor modulating both peripheral ocular processes and central symptom interpretation, thereby contributing to the well-documented discrepancy between objective signs and subjective symptoms in Dry Eye Disease (85). These mechanisms include dysregulation of the hypothalamic–pituitary–adrenal (HPA) axis, sympathetic overactivation, and increased inflammatory signaling, which may contribute to ocular surface inflammation, tear film instability, and heightened sensitivity to corneal nociceptive stimuli. Individuals with high levels of neuroticism are often characterized by increased emotional vigilance and lower discomfort thresholds, which may amplify the perception of dryness, irritation, or fluctuating vision even in the presence of mild or clinically incongruent signs.

Harm avoidance, considered by Kuru et al. (69) together with novelty seeking, may influence coping strategies, by promoting symptom-focused attention, reassurance seeking, or heightened distress. At the biological level, harm avoidance has been linked to increased reactivity of stress-related neurobiological systems, including enhanced amygdala responsiveness and altered serotonergic and autonomic regulation, which are implicated in threat processing and pain sensitivity (87). These alterations may contribute to heightened vigilance toward ocular discomfort and increased sensitivity to corneal nociceptive input. Furthermore, individuals with high harm avoidance tend to adopt avoidant coping strategies, increased symptom monitoring, and anticipatory worry, which may reinforce symptom-focused attention and exacerbate perceived disease burden (88). In Dry Eye Disease, these characteristics may amplify subjective symptom severity independently of objective ocular surface findings, contributing to the discrepancy between clinical signs and patient-reported outcomes. Together, these features suggest that harm avoidance may function as a temperament-based vulnerability factor influencing both central symptom processing and behavioral responses to chronic ocular discomfort. Novelty seeking reflects individual differences in dopaminergic-driven behavioral activation and cognitive flexibility, with lower levels associated with reduced adaptive coping and prolonged focus on aversive bodily sensations (89, 90). In this context, reduced novelty seeking may therefore contribute to difficulty disengaging from ocular discomfort and greater perceived symptom burden, particularly in chronic or fluctuating disease courses.

Another personality-related construct potentially relevant to Dry Eye Disease is frustration tolerance, examined by Kaiser and colleagues (68) and commonly conceptualized within the broader framework of distress tolerance. Distress tolerance refers to an individual’s perceived or actual capacity to withstand aversive internal states, such as discomfort, frustration, or uncertainty, without becoming overwhelmed or engaging in maladaptive coping strategies (91, 92). Low frustration tolerance has been consistently associated with heightened sensitivity to unpleasant bodily sensations, difficulties in emotion regulation, and increased attentional focus on distressing internal cues. Neurobiological and psychological models suggest that distress tolerance relies on effective top-down regulatory control, involving prefrontal systems that modulate limbic and interoceptive responses to persistent discomfort (93, 94). In chronic or fluctuating conditions such as DED, reduced frustration tolerance may limit patients’ ability to adapt to ongoing ocular discomfort, contributing to increased symptom salience, reduced persistence in coping efforts, and greater perceived disease burden. From a behavioral perspective, individuals with low frustration tolerance may exhibit reduced tolerance for symptom variability and uncertainty, thereby amplifying subjective symptom severity and negatively impacting quality of life, even in the presence of relatively stable or mild ocular surface findings.

Two innovative contributions have examined anxiety sensitivity related to DED (72, 73). Anxiety sensitivity refers to the tendency to fear anxiety-related sensations because they are interpreted as harmful, encompassing concerns about physical, cognitive, and social consequences of arousal (95). Higher anxiety sensitivity has been associated with increased attention to and amplification of internal bodily sensations and negative emotional states, which can heighten subjective symptom reporting and distress. Individuals with elevated anxiety sensitivity may therefore perceive Dry Eye Disease symptoms as more intense and frequent compared with those with lower anxiety sensitivity.

Similar associations between personality traits and symptom burden have been reported across other chronic medical conditions, where traits such as neuroticism influence patient-reported outcomes and psychological adjustment, suggesting that the mechanisms observed in Dry Eye Disease may extend beyond ocular pathology (96).

These mechanisms suggest that DED symptoms should not be examined solely through objective ocular parameters, as personality-related stress reactivity may partly mediate the known discrepancy between signs and symptoms in a subset of patients. Across the included studies, neuroticism consistently emerged as a key personality dimension associated with higher symptom burden and reduced quality of life. Nonetheless, the strength of this association may depend on study design. Half of the included studies provided a clinically confirmed diagnosis, whereas the others relied exclusively on self-reported symptom measures. This distinction is methodologically relevant, as personality traits, particularly neuroticism, may influence both symptom interpretation and reporting. To date, however, this potential moderating effect has not been systematically explored.

Further research is needed to clarify the mechanisms through which personality traits shape DED symptom perception and disease burden. Personality dimensions are relatively stable and differ from transient affective states; therefore, future studies should adopt longitudinal designs to examine temporal relationships and directionality. The predominance of cross-sectional studies in the existing literature limits causal inference and reduces the generalizability of the findings. Intervention studies and comparative analyses across clinical subgroups are also lacking. Another limitation concerns the reliance on self-reported measures in half of the included studies, which may introduce subjective bias and compromise diagnostic accuracy. Although validated instruments such as the OSDI and WHS are widely used, clinical evaluation remains essential for accurate diagnosis. Future research should also consider the limitations of commonly used symptom questionnaires such as the Ocular Surface Disease Index (OSDI), which primarily captures symptom frequency and functional impact while provides less detailed information on symptom variability and temporal characteristics. Complementary instruments with greater sensitivity to symptom fluctuations and onset, such as the Standard Patient Evaluation of Eye Dryness (SPEED), may therefore contribute to a more nuanced assessment of Dry Eye Disease symptoms.

Despite these limitations, this review highlights the absence of previous systematic work on personality traits in DED and contributes to defining the current state of evidence. The findings emphasize the importance of considering personality characteristics when assessing patients with DED or DED-related symptoms, suggesting that psychological functioning may meaningfully influence symptom perception, emotional distress, and overall quality of life.

## 5 Conclusion

This systematic review highlights that personality traits are meaningfully associated with both the subjective experience and the clinical burden of Dry Eye Disease. Among these traits, neuroticism consistently emerged as a key dimension linked to greater symptom severity, heightened psychological distress, and reduced quality of life. These findings reinforce the view that DED is not only a disorder of the ocular surface but also a condition influenced by psychological functioning and individual stress reactivity. Furthermore, the perception of DED symptoms may partly reflect increased psychological sensitivity and stress responsiveness related to innate biological predispositions shaping emotional and behavioral responses.

From a clinical perspective, incorporating personality assessment into routine ophthalmologic and psychosomatic evaluation may help identify patients who are particularly vulnerable to symptom amplification, emotional distress, or reduced adherence to treatment recommendations. Recognizing these psychological dimensions could support more refined patient stratification and strengthen the therapeutic alliance, ultimately improving clinical outcomes. Assessing personality alongside other psychological domains may enhance diagnostic accuracy by identifying sub threshold psychological factors that could indicate the need for psychological, rather than exclusively ophthalmologic interventions.

Future research should expand the current evidence base through longitudinal, multimethod, and clinically validated designs, directly comparing individuals with clinically confirmed DED with those presenting only self-reported symptoms. Such approaches are essential to clarify causal pathways, identify potential moderators and mediators, and develop tailored multidisciplinary interventions addressing both ocular and psychological needs.

## Data Availability

The original contributions presented in the study are included in the article/Supplementary material, further inquiries can be directed to the corresponding author.
